# Доклиническая диагностика синдрома фон Хиппеля-Линдау у ребенка грудного возраста

**DOI:** 10.14341/probl13280

**Published:** 2024-02-28

**Authors:** О. А. Малиевский, Р. И. Малиевская, В. А. Малиевский, А. Н. Тюльпаков

**Affiliations:** Башкирский государственный медицинский университет; Башкирский государственный медицинский университет; Башкирский государственный медицинский университет; Медико-генетический научный центр имени академика Н.П. Бочкова; Российская детская клиническая больница

**Keywords:** дети, феохромоцитома, синдром фон Хиппеля-Линдау

## Abstract

Представлено описание случая диагностики синдрома фон Хиппеля-Линдау у ребенка в возрасте пяти месяцев, не имеющего каких-либо проявлений данного заболевания. Поводом для молекулярно-генетического обследования стало наличие случаев данного синдрома в семье (мама и сестра). В гене VHL был выявлен гетерозиготный вариант c.355T>C p.F119L. При объективном обследовании со стороны внутренних органов патологии не выявлено. Комплексное лабораторно-инструментальное обследование, направленное на поиск компонентов синдрома фон Хиппеля-Линдау, в том числе анализ крови на метанефрины и норметанефрины, УЗИ органов брюшной полости, осмотр глазного дна, также не выявило каких-либо отклонений. Учитывая результаты молекулярно-генетической диагностики, ребенок остается под наблюдением и будет проходить регулярное обследование с целью выявления компонентов синдрома фон Хиппеля-Линдау, включая анализы крови/мочи на норметанефрины.

## Abstract (en)

The description of the child aged 5 months with the von Hippel-Lindau syndrome without any manifestations of this syndrome is presented. The reason for the molecular genetic examination was the presence of cases of this syndrome in the family (mother and sister). The heterozygous variant c.355T>C p.F119L was found in the VHL gene. An objective examination revealed no pathology. A comprehensive laboratory and instrumental examination aimed at searching for components of the von Hippel-Lindau syndrome, including a blood test for metanephrines and normetanephrines, ultrasound of the abdominal organs, examination of the fundus, also did not reveal any abnormalities. Given the results of molecular genetic diagnosis, the child remains under observation and will undergo regular examinations to identify components of the von Hippel-Lindau syndrome, including blood/urine tests for normetanephrines.

## АКТУАЛЬНОСТЬ

Феохромоцитомы/параганглиомы (ФХЦ/ПГЛ) — это нейроэндокринные опухоли, развивающиеся из хромаффинных клеток мозгового вещества надпочечников (80–90% случаев) и вненадпочечниковой локализации, выделяющие катехоламины в избыточном количестве. В детском возрасте ФХЦ/ПГЛ характеризуются преобладанием вненадпочечниковой локализации, двусторонним поражением надпочечников в случае надпочечниковой локализации ФХЦ, рецидивирующим течением и мультифокальностью поражения [1][2]. В детском же возрасте ФХЦ/ПГЛ проявляются артериальной гипертензией в 64–93% случаев, головной болью в 39–95%, потливостью в 90%, сердцебиением в 53%, признаками/симптомами масс-эффекта. В 30% случаев заболевание выявляется в виде случайного образования надпочечников [3].

В отличие от взрослой популяции доля моногенных заболеваний, вызывающих развитие ФХЦ/ПГЛ в детском и молодом возрасте, гораздо выше (соответственно 40 и 80%) [1][2]. У детей с ФХЦ/ПГЛ чаще всего наблюдаются мутации в генах, связанных с активацией гипоксияиндуцированного сигналлинга, в частности в гене фон Хиппеля-Линдау (VHL), и в генах, кодирующих одну из субъединиц сукцинатдегидрогеназы (SDH). Синдром фон Хиппеля-Линдау (VHL-синдром) у детей является наиболее частой генетической причиной ФХЦ/ПГЛ [4]. VHL-синдром развивается вследствие инактивирующей герминальной мутации в гене-супрессоре VHL на хромосоме 3p25.3, имеет аутосомно-доминантный тип наследования и высокую пенетрантность (более 90%). Данный ген кодирует две изоформы белка с молекулярными массами 30 кДа (полная форма) и 19 кДа (укороченная форма), играющие важную роль в регуляции реакции на гипоксию и необходимые для поддержания клеточного гомеостаза. VHL опосредует инвазию и метастазирование опухоли путем регуляции экспрессии белка HIFs [5][6]. Примерно у 20% пациентов причиной заболевания являются мутации do novo [7]. Среди герминальных мутаций наиболее распространенными являются миссенс-мутации (50–70%), тогда как нонсенс-мутации и мутации сайтов сплайсинга встречались реже [8][9]. Особенностью течения VHL-синдрома является корреляция генотип–фенотип. В частности показано, что с миссенс-мутациями связаны большие риски метастатических заболеваний и более агрессивное течение с быстрой прогрессией клинических проявлений.

Помимо феохромоцитом, при VHL-синдроме могут развиваться высоковаскуляризированные опухоли, нейроэндокринные опухоли поджелудочной железы, придатка яичка, центральной нервной системы, гемангиобластома сетчатки, карциноидный синдром, кисты в почках и поджелудочной железе, что требует мультидисциплинарного подхода к диагностике, наблюдению за пациентами и их терапии. ФХЦ/ПГЛ развивается в 10–25% случаев VHL-синдрома, чаще отмечаются феохромоцитомы, а риск метастатического заболевания составляет 3–8% [10][11].

## ОПИСАНИЕ СЛУЧАЯ

Ребенок К. был вызван врачом — детским эндокринологом для обследования в связи с наличием у нескольких членов семьи феохромоцитомы.

У мамы — злокачественная феохромоцитома (адреналэктомия справа — в 2001 году, слева — в 2015 году). Молекулярно-генетическая диагностика у нее не проводилась. У родного брата ребенка в возрасте пяти лет при УЗИ надпочечников диагностирована феохромоцитома без каких-либо клинических проявлений, в т.ч. без артериальной гипертензии и тахикардии. На КТ органов брюшной полости в проекции обоих надпочечников выявлены мягкотканные образования, имеющие округлую форму с четкими и ровными контурами размерами справа — 24х13,5х24,5 мм, слева — 23х29,5х37 мм, неоднородной плотности от +35 ед.Н в центре до +55 +57 ед.Н по периферии. В разовой порции мочи уровень фракционированных метанефринов (свободные + конъюгированные) — 65 мкг/г креатинина, фракционированных норметанефринов — 5980 мкг/г креатинина. Была проведена билатеральная адреналэктомия. Гистологические изменения соответствовали феохромоцитоме с высоким метастатическим потенциалом (6 баллов по шкале PASS, 6 баллов по шкале GAPP). В последующем отмечались два локальных рецидива, а также метастазы в легких. В рамках программы «АльфаЭндо» в ФГБУ «НМИЦ эндокринологии» Минздрава России проведено молекулярно-генетическое исследование, в ходе которого в гене VHL при секвенировании по Сэнгеру был выявлен гетерозиготный вариант c.355T>C p.F119L.

У тети по материнской линии ранее также была выявлена двусторонняя феохромоцитома, по поводу которой проведена билатеральная адреналэктомия. Молекулярно-генетическая диагностика не проводилась. У ее дочери феохромоцитома была диагностирована случайно в возрасте 11 лет во время профилактического медосмотра в школе. Во время УЗИ органов брюшной полости в проекции правого надпочечника определялось образование овальной формы с четкими ровными контурами жидкостно-солидной структуры размерами 58х30х40 мм, солидный компонент однородной структуры, средней эхогенности, жидкостный компонент в виде нескольких жидкостных включений размерами 7–22х13 мм. На КТ в области правого надпочечника обнаружено образование овальной формы размерами 41х28х48 мм, плотностью от +21 до +40 ед.Н, с участком неправильной формы, пониженной плотности до +10 ед.Н. Каких-либо клинических проявлений заболевания, в т.ч. артериальной гипертензии, не было. В плазме крови уровень адреналина — 17 пг/мл (норма 18–460), норадреналина — 20629 пг/мл (норма 85–1250), дофамина — 82,6 пг/мл (норма 50–220), серотонина — 26 нг/мл (норма 50–220 нг/мл). Экскреция свободных метанефринов с мочой составила 15,24 мкг/сут (норма 7,69–33,33 мкг/сут), свободных норметанефринов — 2595,87 мкг/сут (норма 7,91–35,18 мкг/сут), норадреналина — 2517 мкг/сут (норма 15–80 мкг/сут), дофамина — 264 мкг/сут (норма 65–400 мкг/сут). В гене VHL также был выявлен гетерозиготный вариант c.355T>C p.F119L.

При обследовании ребенка К. родители каких-либо жалоб на его здоровье не предъявляли. При объективном обследовании со стороны внутренних органов патологии не выявлено. Уровень артериального давления составил 88/54 мм рт. ст., частота сердечных сокращений 112 ударов в минуту. Отсутствовали симптомы избытка катехоламинов в виде потливости, артериальной гипертензии, тахикардии, полиурии. Голову удерживает с полутора месяцев, сидит с поддержкой. Отсутствовала очаговая неврологическая симптоматика, симптомы поражения черепных нервов, патологические рефлексы. Взгляд фиксирует, узнает родителей и членов семьи, что свидетельствует об отсутствии нарушения зрения.

Учитывая отсутствие артериальной гипертензии и тахикардии у сибса, имеющего феохромоцитому, проведено исследование уровня метанефринов и норметанефринов в плазме крови, хотя эксперты Alliance VHL рекомендуют делать это после достижения 5-летнего возраста. Уровень свободного метанефрина в плазме крови составил 0,25 нмоль/л (норма 0–0,49 нмоль/л), свободного норметанефрина — 0,57 нмоль/л (норма 0–0,89 нмоль/л), что позволило исключить феохромоцитому. Комплексное объективное и лабораторно-инструментальное обследование, направленное на поиск компонентов синдрома фон Хиппеля-Линдау, включающее осмотр глазного дна, УЗИ органов брюшной полости и почек, не выявило каких-либо отклонений. В гене VHL был выявлен гетерозиготный вариант c.355T>C p.F119L. Учитывая результаты молекулярно-генетической диагностики, ребенок остается под наблюдением и будет проходить регулярное обследование с целью выявления компонентов синдрома фон Хиппеля-Линдау, включая анализы крови/мочи на норметанефрины.

## ОБСУЖДЕНИЕ

Представленное описание клинического наблюдения свидетельствует о возможности доклинической диагностики наследственных форм феохромоцитом путем проведения молекулярно-генетического обследования взрослых пациентов и членов их семей. Это позволит выяалять как развитие феохромоцитомы, так и других компонентов VHL-синдрома на ранней стадии, избежать тем самым их метастазирования и улучшить прогноз для жизни пациентов [2]. Необходимость активного выявления детей с семейными формами феохромоцитом обосновывается тем, что достаточно часто они бывают бессимптоными. Выявленный у нашего пациента патогенный вариант c.355T>C p.F119L относится к миссенс-мутациям, которые являются наиболее частыми и с которыми связаны большие риски метастатических заболеваний и более агрессивное течение с быстрой прогрессией клинических проявлений [4][9]. Таким образом, знание типа мутации позволяет не только выявлять VHL-синдром до развития феохромоцитомы и каких-либо других клинических проявлений, но и прогнозировать течение феохромоцитомы и, возможно, определять тактику лечения. В исследовании Michael Reich показано, что вероятность возникновения различных компонентов VHL-синдрома увеличивается с возрастом [12]. Это обосновывает необходимость постоянного наблюдения за пациентами мультидисциплинарной командой.

Вариант c.355T>C p.F119L был описан ранее у пациентов с различными проявлениями VHL-синдрома. Kang с соавт. выявили данную мутацию у пациента с опухолью ЦНС и гемангиобластомой сетчатки и без наличия феохромоцитомы [14], тогда как в серии Albattal с соавт. данная мутация описана у пациента с двусторонней феохромоцитомой без других компонентов синдрома [15]. Лица, у которых при ДНК-анализе не выявлено изменений в гене VHL, не нуждаются в постоянном наблюдении. Для прогнозирования относительного риска определенных проявлений у членов семьи, имеющих данный синдром, выделяют несколько категорий VHL-синдрома. Эти категории определяются типом мутации и характеризуются высоким или низким риском различных проявлений синдрома в зависимости от мутации. Несмотря на это, всем пациентам с VHL-синдромом рекомендуется наблюдение по всем его возможным признакам, независимо от подтипа [13].

В соответствии с рекомендациями экспертов по VHL-синдрому (VHL Alliance, 2020 год) наблюдение за детьми необходимо начинать уже в грудном возрасте [13]. Лицам молодого возраста требуется офтальмологическое обследование, включая осмотр глазного дна. VHL Alliance рекомендует «правило 5–11–15»: с 5 лет ежегодно исследование суточной мочи или анализ крови на норметанефрины и метанефрины, с 11 лет сурдологическое обследование и томография головного и спинного мозга каждые два года, с 15 лет — МРТ брюшной полости каждые два года (табл. 1).

**Table:** Таблица 1. Рекомендации по активному наблюдению за пациентами с VHL-синдромом (VHL Alliance, 2020 год) [13]

Вид обследования (скрининг опухолей)	Возраст	При беременности
До 5 лет	С 5 лет	С 11 лет	С 15 лет	С 30 лет	С 65 лет
Анамнез и осмотр	Ежегодно с 1 года	Ежегодно	Ежегодно	Ежегодно	Ежегодно	Ежегодно	До зачатия
АД и пульс (феохромоцитома, параганглиома)	Ежегодно с 2 лет	Ежегодно	Ежегодно	Ежегодно	Ежегодно	Ежегодно	До зачатия
Глазное дно (ретинальная гемангиобластома)	Каждые 6–12месяцев начиная с 1 года	Каждые6–12месяцев	Каждые6–12месяцев	Каждые6–12месяцев	Ежегодно	Ежегодно	До зачатия, а затем каждые6–12месяцев
Метанефрины (феохромоцитома, параганглиома)		Ежегодно	Ежегодно	Ежегодно	Ежегодно	Закончить обследование	До зачатия
МРТ ЦНС с/без контраста (гемангиобластома ЦНС)			Каждые 2 года	Каждые 2 года	Каждые 2 года	Закончить обследование	До зачатия
Аудиограмма (опухоль эндолимфатического мешка)			Каждые 2 года	Каждые 2 года	Каждые 2 года	Закончить обследование	
МРТ брюшной полости с/без контраста (почечноклеточный рак, феохромоцитома/параганглиомы, нейроэндокринные опухоли/кисты поджелудочной железы)				Каждые 2 года	Каждые 2 года	Закончить обследование	До зачатия
МРТ внутренних слуховых ходов (опухоль эндолимфатического мешка)				Однократно			

В «Руководстве для людей с болезнью фон Хиппеля-Линдау, их семей и медицинских профессионалов» VHL Alliance при VHL-синдроме рекомендуется использовать МРТ вместо КТ, чтобы снизить общее воздействие радиации на людей с VHL-синдромом в течение жизни. Чтобы контролировать особенно важные области головного и спинного мозга, наиболее эффективным и экономичным способом является МРТ ЦНС, включая МРТ головного мозга, шейного, грудного и поясничного отделов позвоночника. Чтобы исключить гемангиобластомы головного и спинного мозга, необходимо выполнять сканирование на аппарате МРТ с мощностью магнитного поля не менее 1,5 Тл (Тесла) с контрастным усилением или без него и обязательно с тонкими срезами области задней черепной ямки [13]. При проведении МРТ ЦНС предпочтительно использовать макроциклические контрастные вещества и контрастные вещества на основе гадолиния класса II.

Помимо общепринятых сбора анамнеза и физикального обследования, у пациентов проводятся неврологическое обследование, слуховые и вестибулоневральные тесты, оценка симптомов избытка катехоламинов (головные боли, сердцебиение, потоотделение, гиперактивность, беспокойство, полиурия, боли в животе).

Расширенное офтальмологическое обследование, включая офтальмоскопию, с целью исключения гемангиобластомы сетчатки должно проводиться каждые 6–12 месяцев в зависимости от результата проведенного ранее обследования (особенно у детей) и предполагаемой необходимости последующего наблюдения. Если невозможно провести подробное офтальмологическое обследование обычным способом, необходимо рассмотреть возможность обследования детей под наркозом. Также целесообразно оценить возможность выполнения ультраширокоугольного фотографирования и ультраширокоугольной флюоресцентной ангиографии, которые не должны заменять осмотр офтальмолога.

Для диагностики феохромоцитомы целесообразно определять уровень свободных метанефринов плазмы в силу их более высокой чувствительности по сравнению с измерением уровня метанефринов в суточной моче [13].

МРТ органов брюшной полости направлена на раннее выявление кист и карциномы почек, серозных цистаденом и нейроэндокринных опухолей поджелудочной железы, ФХЦ/ПРГ надпочечников и вненадпочечниковой локализации. Она может быть выполнена одновременно с МРТ головного мозга в рамках одного длительного наркоза, особенно у детей. Однако протоколы МРТ ЦНС и брюшной полости следует выполнять последовательно. Не рекомендуется оценивать изменения позвоночника с помощью протокола МРТ брюшной полости или оценивать состояние органов брюшной полости, используя результаты протокола МРТ ЦНС. Если гемангиобластомы ЦНС не выявлены, плановое наблюдение продолжают каждые 2 года. При наличии гемангиобластом(-ы) и увеличении их (ее) размеров и (или) при наличии у пациента сопутствующих симптомов сканирование следует проводить ежегодно (или чаще), в зависимости от ситуации (или направлять к нейрохирургу). Если при первоначальном сканировании не обнаружено поражений почек, продолжают плановое наблюдение каждые 2 года. Если обнаружены небольшие опухоли почек (менее 3 см), то повторяют МРТ каждые 3–6 месяцев до определения стабильности. После того как стабильность будет определена в течение трех последовательных сканирований, рассматривают возможность повторных исследований — один раз каждые 2 года. Если почечное новообразование 3 см и более, рекомендуется консультация уролога. Магнитно-резонансную томографию с высоким разрешением (толщина среза 1 мм) для исследования внутреннего слухового прохода следует проводить после 15 лет [13].

У лиц старше 65 лет, у которых к этому возрасту не было выявлено никаких проявлений VHL-синдрома, регулярное медицинское и офтальмологическое обследования целесообразно продолжить [13].

## ЗАКЛЮЧЕНИЕ

К сожалению, в описанных нами семьях ни одному из взрослых пациентов не была предложена молекулярно-генетическая диагностика, хотя согласно клиническим рекомендациям Российской ассоциации эндокринологов по диагностике и лечению феохромоцитомы/параганглиомы всем пациентам с ФХЦ/ПГ рекомендуется проведение генетического обследования [2]. Необходимость молекулярно-генетической диагностики объясняется тем, что более трети всех пациентов с ФХЦ/ПГ имеют наследственные мутации, диагностирование наследственного синдрома у пробанда является залогом своевременной диагностики и лечения других членов семьи, а выявление генетического дефекта создает возможность использования предимплантационной и пренатальной диагностики. Законным представителям обследованных нами детей были разъяснены цели и результаты генетического тестирования, важность обследования всех прямых родственников. У ребенка с молекулярно-генетически подтвержденным VHL-синдромом составлен план длительного наблюдения с учетом рекомендаций VHL Alliance.

## ДОПОЛНИТЕЛЬНАЯ ИНФОРМАЦИЯ

Источники финансирования. Работа выполнена по инициативе авторов без привлечения финансирования.

Конфликт интересов. Авторы декларируют отсутствие явных и потенциальных конфликтов интересов, связанных с содержанием настоящей статьи.

Участие авторов. Все авторы внесли существенный вклад в получение, анализ и интерпретацию результатов, поиск и оценку данных литературы, написание статьи или внесение в рукопись существенных (важных) правок с целью повышения научной ценности статьи. Все авторы одобрили финальную версию статьи перед публикацией, выразили согласие нести ответственность за все аспекты работы, подразумевающую надлежащее изучение и решение вопросов, связанных с точностью или добросовестностью любой части работы.

Согласие пациента. Законный представитель пациентов добровольно подписал информированное согласие на публикацию персональной медицинской информации в обезличенной форме в журнале «Проблемы эндокринологии».
